# Development and Validation of the Keele Musculoskeletal Patient Reported Outcome Measure (MSK-PROM)

**DOI:** 10.1371/journal.pone.0124557

**Published:** 2015-04-30

**Authors:** Jonathan C. Hill, Elaine Thomas, Susan Hill, Nadine E. Foster, Danielle A. van der Windt

**Affiliations:** Institute of Primary Care and Health Sciences, Keele University, Stoke-on-Trent, Staffordshire, United Kingdom; University of Texas Health Science Center at Houston, UNITED STATES

## Abstract

**Objective:**

To develop and validate a patient report outcome measure (PROM) for clinical practice that can monitor health status of patients with a range of musculoskeletal (MSK) disorders.

**Methods:**

Constructs for inclusion in the MSK-PROM were identified from a consensus process involving patients with musculoskeletal conditions, clinicians, purchasers of healthcare services, and primary care researchers. Psychometric properties of the brief tool, including face and construct validity, repeatability and responsiveness were assessed in a sample of patients with musculoskeletal pain consulting physiotherapy services in the United Kingdom (n=425).

**Results:**

The consensus process identified 10 prioritised domains for monitoring musculoskeletal health status: pain intensity, quality of life, physical capacity, interference with social/leisure activities, emotional well-being, severity of most difficult thing, activities and roles, understanding independence, and overall impact. As the EuroQol (EQ-5D-5L) is a widely adopted PROMs tool and covers the first four domains listed, to reduce patient burden to a minimum the MSK-PROM was designed to capture the remaining six prioritised domains which are not measured by the EQ-5D-5L. The tool demonstrated excellent reliability, construct validity, responsiveness and acceptability to patients and clinicians for use in clinical practice.

**Conclusion:**

We have validated a brief patient reported outcome measure (MSK-PROM) for use in clinical practice to measure musculoskeletal health status and monitor outcomes over time using domains that are meaningful to patients and sensitive to change. Further work will establish whether the MSK-PROM is useful in other musculoskeletal healthcare settings.

## Introduction

Patient Reported Outcome Measures (PROMs) are defined as “… any report of the status of a patient’s health condition that comes directly from the patient, without interpretation of the patient’s response by a clinician or anyone else”[1]. Appropriate PROM tools for longer-term, fluctuating musculoskeletal disorders, are particularly important as their clinical management is primarily focused on addressing symptoms rather than laboratory results or biomarkers. The need for PROMs that are primarily designed for clinical practice rather than research is also increasing in order to place patients’ needs, interests and feedback at the centre of care[2–4]. PROMs are recognised for their role as catalysts for organisational change both through their use at an aggregate level (e.g. audits)[3,4], and individual level (e.g. helping patients to monitor their own health)[5]. Momentum among policy makers is growing for the collection of PROMs data in order that clinical services can provide standardised reports of their clinical outcomes[6]. An example which reflects this trend within musculoskeletal healthcare is the UK’s National Health Service Programme for PROMs (http://www.ic.nhs.uk/proms) which aims to raise healthcare standards by reporting provider performance for high-cost surgical procedures including hip and knee joint replacement.

Outcome measures can be categorised into those which are ‘condition specific’ and those which are ‘generic’ and are applicable across different health conditions. The EuroQol (EQ-5D)[7] is a high profile PROM used to evidence outcomes of care as it is ‘generic’ and enables comparisons of health status improvements across different patient populations[6]. Its scores can also be converted into ‘utility estimates’ that are used in health economic evaluations[2,7]. The EQ-5D has five items; mobility, self-care, usual activities, pain and emotional health, each with three response options and has recently been superseded by the EQ-5D-5L[8] which offers five response levels to reduce ceiling effects and improve discriminatory power[9]. The EQ-5D-5L has therefore been recommend for clinical use within musculoskeletal (MSK) populations and has been adopted by the UK National PROMs Programme and professional organisations including the Chartered Society of Physiotherapy (www.csp.org.uk/proms). In addition to generic PROMs clinical services may also collect condition specific outcomes with greater relevance and responsiveness to their particular patient population, such as the Roland Morris Disability Questionnaire for low back pain[10], or the Oxford Knee Score for knee osteoarthritis[11]. However, capturing condition specific data for multiple musculoskeletal disorders can be difficult in routine practice and clinicians have therefore called for more feasible, simple yet fit for purpose PROM tools which can assess overall health status of patients with MSK pain different body regions (e.g. knee, shoulder and neck). At present numerous lengthy research instruments are available for musculoskeletal disorders that are specific to regions of the body and that measure individual constructs (e.g. the Roland Morris Disability Questionnaire has 24 items to capture physical function in people with low back pain). However, clinicians and patients do not find such research measures practical for routine clinical care and self-monitoring purposes, particularly since they are perceived as too long for use in clinical practice and because many patients have more than one musculoskeletal pain problem [12,13]. Instead new tools are needed that can be applied across a range of common musculoskeletal disorders, which cover a range of different constructs, and are designed for use in clinical practice and therefore and brief, simple to use and interpret. Such a tool does not seek to replace research measures, but to enable routine objective clinical assessment of key constructs that are pertinent to a particular patient population, ie. those with common musculoskeletal disorders. Important requirements for such a musculoskeletal PROM would be to; include priority health outcomes for musculoskeletal patients, be easily interpretable and feasible for use in busy clinical practice, provide excellent reliability and superior responsiveness to existing measures used for this purpose, such as the EQ-5D-5L.

In this study we therefore aimed to develop and validate a brief musculoskeletal (MSK) pain-specific PROM (MSK-PROM) suitable for clinical practice. The specific objectives were: 1) to prioritise the primary outcomes of treatment for musculoskeletal disorders with stakeholders, including patient and clinician representatives, 2) to develop an MSK-PROM to monitor these outcomes with strong face and content validity, 3) to investigate test-retest reliability, convergent construct validity and responsiveness of the candidate MSK-PROM compared to EQ-5D-5L, and 4) to examine its feasibility and acceptability for use in routine clinical consultations.

## Methods

### Objective 1: Prioritising outcome domains for musculoskeletal disorders

#### Consensus workshops

Two iterative consensus workshops were held with regional stakeholders (including musculoskeletal; patients, clinicians, researchers, service managers and purchasers) to agree and prioritise treatment targets/outcomes for MSK-PROM inclusion. All participants provided informed written consent and were remunerated according to INVOLVE recommendations[14]. A nominal group technique[15] consensus process was used involving: a study presentation, small group discussions (including a dedicated patient group) to identify potential health domains for inclusion, a full group discussion, a blind vote to retain domains with broad consensus (defined as >50% of participants), and finally individual participant ranking of domains. Patient perspectives were specifically prioritised throughout this process. Participants attended a second workshop two weeks later to further discuss and revise the list of ranked domains before re-ranking them again.

#### A national stakeholder consultation

An on-line survey then established if the health domains identified by the regional group of stakeholders were considered a priority nationally. Participants were recruited through advertisments in national stakeholder forums and charitible organisation websites including Back Care, the Arthritis Rheumatism Musculoskeletal Alliance, Arthritis Care and the Chartered Society of Physiotherapy’s interactive online forum—iCSP and the Physiotherapy Consultant’s Forum. The survey sought participant consent, identified if the particpant was a health professional or patient, and then presented a list of the top ten ranked health domains for musculoskeletal disorders alongside a separate list of identified domains that were not ranked within the top ten. Participants were asked four questions: i) if they agreed with domains on the top ten list, ii) if they agreed with the ranking of constructs, iii) whether other important health domains were missing, and iv) if they had any additional comments.

The results were collated and examined by the research team including two patient representatives.

### Objective 2: Developing the MSK-PROM

Having prioritised key musculoskeletal outcomes, single items for each domain were formulated during two interactive face validity workshops with five members of the Research Institute’s established musculoskeletal Research User Group and two experienced musculoskeletal clinical researchers. This process generated an item for each domain that was clear to users and had appropriate and optimal content validity. Domains already captured by the EQ-5D-5L were examined to avoid unneccessary duplication with the MSK-PROM. The readability and comprehensibility of tool items, instructions and response options were examined. The culmination of this process was a draft MSK-PROM ready for psychometric testing.

### Objective 3: Measurement properties of the MSK-PROM

#### Design and setting

A prospective cohort study was conducted among out-patient musculoskeletal physiotherapy clinics in five UK West Midlands towns (Congleton, Cannock, Shrewsbury, Oswestry, Cheadle). These clinics provide individual, face-to-face treatments within the UK National Health Service (NHS) with most patients accessing care following a referral from their General Practitioner (GP) or hospital specialist. Participants received usual physiotherapy care according to clinical need.

#### Patient selection

Consecutive adult (> = 18 years) consulters with a musculoskeletal disorder were invited to participate having received a study information pack with their clinic appointment. No further inclusion/exclusion criteria were used. Consenting participants were also asked for further consent to receive an invitation to a post-study feedback workshop to discuss their experiences of using the MSK-PROM.

#### Population descriptors

Prior to initial clinical assessment within the physiotherapy clinic, participants completed a questionnaire containing baseline population descriptors (**see** S1 Text **—Musculoskeletal Case-Mix Descriptors**) including demographic data (age, gender, work status) and patient/pain characteristics: pain related days off work over past three months, referral source/clinician, site of main musculoskeletal problem (collapsed into upper limb, lower limb, spinal, or multi-site pain), pain episode duration, number of pain related visits to their GP in past 3 months, outcome expectations (using a numerical response scale from 0 ‘it will get worse’ to 10 ‘it will be cured’), a single pain catastrophising item from a validated screening tool for back pain patients (the STarT Back Tool[16]) ‘Do you feel that your problem is terrible and that it is never going to get any better?’ using a numerical response scale (0 ‘completely disagree’ to 10 ‘completely agree’), and self-rated general health using the SF-36 item[17] on a scale of 0 ‘poor health’ to 100 ‘perfect health’).

#### Outcome measures

Outcome measures were collected before the start of treatment at each visit and at three-month follow-up using paper questionnaires containing the MSK-PROM and EQ-5D-5L[8]. The EQ-5D-5L utility score was calculated using the UK Crosswalk value set[8], and the sum score (range 5–25) simply summed responses for all five items (from 1 ‘no problems’ to 5 ‘unable/extrem). To ensure simplicity of the MSK-PROM scoring, which emerged as important during the consensus workshops, scores from all six items are summed together (responses coded from ‘never’ = 1 to ‘all the time’ = 5) providing a range from 6–30. The MSK-PROM overall score is not a score of a single construct (reflective model), but a sum of items from six different domains measuring overall musculoskeletal health status (formative model). In line with methodology guidelines for multi-dimensional instruments, internal consistency of the MSK-PROM overall score was therefore not examined.[18] Patient global rating of improvement, a recommended core outcome in chronic musculoskeletal and osteoarthritis trials[19,20] was captured at all follow-up visits (up to five times) and three-month questionnaire. The item asked “Overall compared to the start of treatment, my symptoms are: much better, better, same, worse, or much worse”. Postal data collection at three-month follow-up was used to reduce attrition bias[21], provide a standardised end-point, and ensure follow-up of those attending for just one physiotherapy visit.

#### Statistical analysis and sample size

MSK-PROM and EQ-5D scores were not calculated for participants with any missing baseline data. However, for those who responded and completed their follow-up questionnaires, incidental missing data for individual MSK-PROM items were imputed using the last observation carried forward method if two or less MSK-PROM items were missing[22]. This approach has been shown to be valid particularly in situations where repeated data time-points are available for the same individual [22]. A sensitivity analysis was conducted to test if the results were similar when using complete cases only. MSK-PROM response rates and floor/ceiling effects (<10% of lowest (6) or highest (30) scores) were explored through descriptive analysis.

To examine test re-test reliability among ‘stable’ patients, we used a sample of those who reported they were “the same” on the global improvement item at the second visit (typically after two weeks). Based on previous cohort data this was estimated to be 30% of patients[16]. Patients completed the MSK-PROM in the same clinical setting at both their first and their second visit in order to examine test re-test reliability. An intraclass correlation coefficient (ICC—based on a two-way random effect model) tested the overall MSK-PROM score reliability with the ICC considered acceptable/good when above 0.70, and a weighted Cohen’s Kappa tested item-by-item agreement[18].

To examine the convergent construct validity of the MSK-PROM and EQ-5D-5L, the Pearson correlation between raw sum scores of both instruments at baseline was calculated[18] and to illustrate the relationship between both scale’s distributional characteristics, a boxplot was produced.

To examine responsiveness (sensitivity to change) we calculated the Area Under the Receiver Operating Characteristic Curve (AUC) for discriminating any improvement, from no change or deterioration on the external anchor measured by the global improvement question at three-month follow-up[18]. The AUC for the MSK-PROM alone was compared to that for the EQ-5D-5L and in addition to a combined variable which summed both tools together (score range of 11–55). In sensitivity analyses, the effect size[23] and standardised response mean[24] were also reported. The effect size is the mean change in tool score divided by the standard deviation of baseline score, and the standardised response mean is the mean change in score divided by the standard deviation of mean change (a higher value indicates higher responsiveness for both). The standard error of measurement was calculated as an indicator of error variation in this population, using baseline score standard deviation multiplied by the square root of 1-ICC.

The sample size (n = 425) for the validation cohort was calculated from the minimum number of patients recommended to investigate MSK-PROM; a) responsiveness[25] at three-month follow-up (n = 150) with an estimated three-month postal questionnaire response rate of 50%, and b) reliability among stable patients at the second physiotherapy visit with an estimated 20% loss to follow up of patients between first and second visits and 30% reporting stable symptoms. Using the Donner & Eliasziw[26] approach for estimating sample size for reliability testing we calculated that 102 people were needed to detect a minimum acceptable ICC of 0.70, assuming a true ICC of 0.80, with a power of 80% and 5% significance level.

### Objective 4: MSK-PROM’s feasibility and acceptability for clinical practice

Following completion of the cohort study, a two hour structured feedback workshop was held at the Research Institute with patient and clinician participants from the cohort to discuss the acceptability and feasibility of the MSK-PROM in clinical practice and to agree any modifications required. In addition, participants were asked to discuss potential mis-uses of the measure. Two clinicians from each clinic were invited (a total of 10) and a random selection of participants were invited to ensure that 1 patient agreed to attend from each clinic, using a sequence formed by a random number generator to reduce selection bias.

## Results

### Objective 1: Prioritising outcome domains for musculoskeletal disorders

#### Consensus workshops

The two workshops were attended by four patients, six clinicians, six researchers, four clinical managers and one purchaser of care. The list of 10 prioritised domains is provided in Table 1 and, in ranked order, included: pain intensity, severity of the one thing that is most difficult, understanding about how to deal with symptoms, physical function, quality of life, work interference, independence, ability to do activities and roles that matter, interference with social/leisure activities, and overall impact. Patients and clinicians did not have strongly different domain preferences, but largely agreed with each other. For practical reasons to limit the burden on patients, participants suggested a cap of around 10 domains should be prioritised for inclusion in the PROM tool. However, due to the rankings domains 11–13 were also integrated into the final PROM.

**Table 1:** Identified health domains for musculoskeletal disorders (in ranked order).

Top 10 health domains in ranked order: [rank score]
1. Pain intensity * [323]
2. Severity of the thing that is most difficult [280]
3. Ability to self-manage (understanding how to deal with the symptoms by yourself) _a_ [273]
4. Physical function * [254]
5. Quality of life * [251]
6. Work interference [234]
7. Independence without help of others [224]
8. Ability to do activities/roles that matter [194]
9. Interference with social/leisure activity *[191]
10. Overall impact (bothersomeness) [167]
**Important health domains with consensus but not ranked in the top 10:**
11. Anxiety/worry (feelings of worry) * _a_ [166]
12. Mood /depression * _a_ [160]
13. Difficulties with sleep [154]
14. Fatigue (lack of energy) [120]
15. Knowledge of condition _a_ [119]
16. Fear of physical activity harm [116)
17. Ability to cope with symptoms [85]

* Domains already included in the EQ-5D-5L

^a^ The online consultation survey highlighted importance of ‘anxiety’ and ‘mood’ domains, and patient’s ‘Knowledge of condition’ was merged with domain 3 ‘ability to self-manage’

#### National consultation survey

The online survey was completed by 80 respondents (35 [44%] patients and 45 [56%] clinicians) and confirmed the importance and relevance of the domains with over two-thirds agreeing with the ranking (71%). There were 23 (29%) respondents who disagreed with the ranking and their comments are provided in S2 Text **—Survey Feedback Comments**. In summary, based on survey responses, anxiety and depression were added to the priority list, but no other suggestions were added for practical reasons to keep the tool short and because additional domains had lower ranking scores. In addition, some patients were confused by the term ‘self-manage’ and suggested ‘understanding about how to deal with symptoms by yourself’ was similar to ‘Knowledge of condition’. Therefore, these two domains were merged together giving a final list of 11 prioritised outcome domains.

### Objective 2: Developing the MSK-PROM

During the face validity workshops with five patient representatives it was agreed that any prioritised domains that were already included in the EQ-5D—so pain intensity, physical capacity (using walking and dressing items), quality of life, interference with social and leisure activities and anxiety/mood) had appropriate content validity and would therefore not be needed within the MSK-PROM. This means that the MSK-PROM needs to be used alongside the EQ-5D-5L to capture all prioritised domains, and only needs to capture the six remaining prioritised health domains not covered by the EQ-5D-5L; overall impact using a modified bothersomeness question[27]; work interference using a new item loosely based on the SF-36[17] work item; and new items for severity of the one thing that’s most difficult, understanding about how to deal with the condition, independence, and ability to do activities and roles that matter (**See** S3 Text **—The Keele MSK-PROM**). Patient representatives preferred response options that expressed ‘frequency’ (‘how often’) rather than ‘severity’ (‘how much’) due to the fluctuating nature of their symptoms, and wanted the results of their previous visit entries to appear on the same page to assist self-monitoring as recommended for monitoring tools[28,29]. On average the MSK-PROM took around one minute to complete (similar to the EQ-5D-5L). The Flesch-Kincaid reading grade level of the MSK-PROM is 4.7 which suggests it is “very easy to read”. Patient representatives confirmed they were satisfied with the content validity of the MSK-PROM in capturing the domains identified and that it had appropriate readability and was easy to understand.

### Objective 3: Measurement properties of the MSK-PROM

There were 425 musculoskeletal patients who consented to participate in the cohort study from a potential pool of 1038 new (incident) patients over a three-month recruitment period (41% participation rate). Patients accessed physiotherapy predominantly following a GP referral (75%) although 19% were referred from a hospital specialist, 3% from Accident and Emergency, 1% from self-referral, and 2% were referred from other clinics. Baseline population characteristics, summarised in Table 2, showed a mean age of 53 years (SD 15.2, range 18–94) with 64% female. The most common region of musculoskeletal pain was the low back (23.3%) and the median pain episode duration was seven months (IQR 3–24). At the second physiotherapy visit 339 (79.8%) completed the MSK-PROM, and 225 patients (53%) returned a completed, postal, three-month follow-up questionnaire.

**Table 2:** Patient characteristics at baseline.

Patient characteristics	Total patient number = 425
Age (years)	53.3 (SD 15.2, range 18–94)
Sex, female (n = 418)	271 (63.8%)
Work status (n = 391)	
No time off work	146 (4.4%)
Retired	124 (9%)
Time off in past 3 months, now back at work	44 (10.4%)
Not in work due to health problems	35 (8.2%)
Not in work, not due to health problems	23 (5.4%)
Currently on sick leave	19 (4.5%)
Referral source (n = 416)	
General practitioner (GP)	312 (73%)
Hospital specialist	78 (18.4%)
Accident and emergency	13 (3.1%)
Other health professional	9 (2.1%)
Self-referral	4 (0.9%)
Site of main musculoskeletal problem (n = 424)	
Back	98 (23.1%)
Multi-site pain	83 (19.6%)
Shoulder	59 (13.9%)
Knee	47 (11.1%)
Neck	41 (9.7%)
Ankle/foot	40 (9.4%)
Hip	27 (6.4%)
Other	13 (3.1%)
Wrist	8 (1.9%)
Elbow	5 (1.2%)
Hand	3 (0.7%)
Visited GP for musculoskeletal disorder in past 3 months? Y/N	395 (93%)
If Yes, mean number of visits in past 3 months to GP	1.6 (SD 1.3)
Episode duration (months) *median (n = 370)	7 (IQR 3–24)
Outcome expectation of physiotherapy (0 low -10 high) (n = 418)	7.5 (SD 1.8)
Pain catastrophising (0 low—10 high) (n = 422)	3.8 (SD 3.0)
General health rating (0 low—100 high) (n = 424)	75.3 (SD 19.9)
Number completing MSK-PROM (proxy for attending clinic)	
Visit 1 (baseline)	417 (98.0%)
Visit 2	339 (79.8%)
Visit 3	270 (63.5%)
Visit 4	177 (41.6%)
Visit 5	101 (23.8%)
Visit 6	65 (15.3%)
Returned 3 month MSK-PROM follow-up	225 (52.9%)
Any MSK-PROM follow-up visit and/or three-month follow-up	364 (85.6%)

#### Completion rates

**Fig 1:**
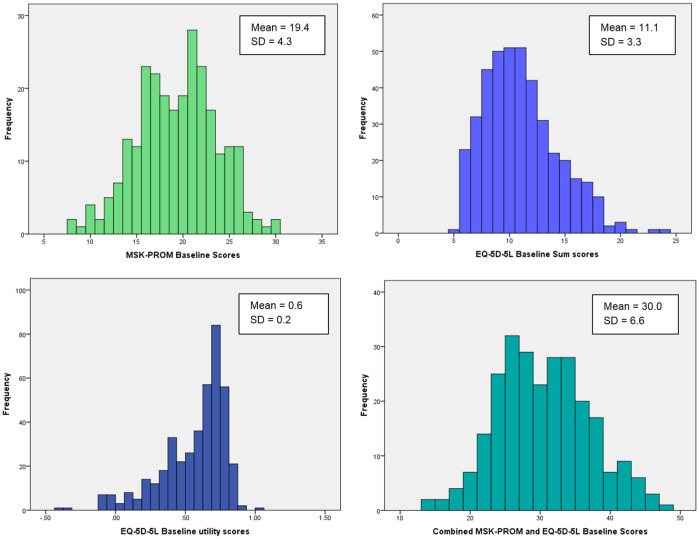
The distribution of mean scores at first visit for the MSK-PROM, EQ-5D-5L sum score and utility score, and a combined MSK-PROM and EQ-5D-5L score.

Complete MSK-PROM and EQ-5D-5L baseline data were available for 417/425 patients (98.1%). The best completed MSK-PROM item was ‘overall impact’ with no missing data and the work item had the most with six missing responses (1.4%) and 158 (37%) ‘not applicable’ responses (due to participants not being in paid work). Due to the high proportion of ‘not applicable’ responses, this item was modified to include ‘daily routine’ to ensure applicability to all patients. The scoring distribution means and SDs for MSK-PROM, EQ-5D-5L sum score and utility score, and a combined MSK-PROM and EQ-5D-5L sum score for first visit data are presented in Fig 1. No weighting was given to any items in order to ensure that the MSK-PROM is simple to use and interpret in clinical practice.

#### Test-retest reliability

There were 129/339 patients (38.0%) reporting ‘stable’ health status between baseline and their second physiotherapy visit (with a 14 day mean time interval) with 41.5% better and 7.4% worse. Within this stable sample the 6-item MSK-PROM sum score ICC was 0.98 (95% CI .97–.99) demonstrating ‘excellent’ reliability and comparable to the EQ-5D-5L sum score ICC of 0.99 (95%CI 0.99, 0.99). The weighted Cohen’s Kappa item-by-item agreement for the MSK-PROM items ranged from 0.96 (95%CI 0.93, 0.98) for ‘work interference’ to 0.82 (95%CI 0.73, 0.90) for ‘severity of the most difficult thing’. The number of MSK-PROM items at the second visit with incidental missing data was: independence = 13, severity of worst problem = 15, work = 12, activities = 13, Understanding = 11, Bothered = 12. The sensitivity analysis using complete data for test re-test showed similar results with an ICC of 0.98 (95% CI .96–.98).

#### Convergent construct validity

**Fig 2:**
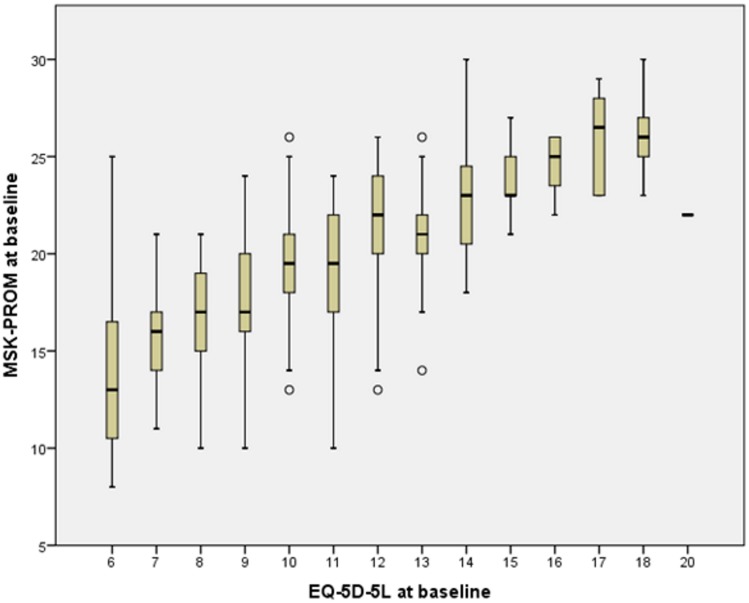
Boxplot presenting the distribution of MSK-PROM and EQ-5D-5L baseline sum scores.

The Pearson correlation of the MSK-PROM and EQ-5D-5L was 0.671 and boxplot illustrating the distribution characteristics of both tool scores is presented in Fig 2. The results demonstrate strong convergent construct validity between the MSK-PROM and the EQ-5D-5L.

#### Responsiveness (sensitivity to change)

Data at three-month follow-up were available for 225/425 (54%) of participants. Non-responders at three-month follow-up were younger (10 year mean difference) and had slightly lower MSK-PROM scores at baseline (1.4 mean lower) compared to responders, but were similar in other baseline characteristics. Among responders, the mean (SD) MSK-PROM score was 15.41 (4.93), and the mean (SD) change from baseline was 3.97 (4.84), with 74.5% reporting better, 5.9% the same and 19.6% worse scores. The responsiveness statistics of the MSK-PROM are presented in Table 3. The MSK-PROM standard error of measurement was 0.86.

**Table 3:** Responsiveness of the MSK-PROM compared to the EQ-5D-5L.

	MSK-PROM	EQ-5D-5L Utility score	EQ-5D-5L Raw sum score	Combined MSK-PROM & EQ5D-5L score
Baseline (n = 415) Mean (SD)	19.35 (4.28)	0.56 (0.23)	11.05 (3.34)	29.9 (6.64)
Three-month follow up (n = 225 FU) Mean (SD)	15.41 (4.93)	0.67 (0.22)	9.450 (3.55)	23.87 (7.36)
Baseline to three-month change—Mean (SD)	3.97 (4.84)	-0.09 (0.22)	1.41 (3.21)	6.20 (7.13)
AUC (95% CI)	0.81 (0.72, 0.90)	0.71 (0.63, 0.79)	0.74 (0.64, 0.85)	0.81 (0.72, 0.90)
Effect size	0.93	0.39	0.42	0.93
Standardised response mean	0.82	0.41	0.44	0.87

### Objective 4: MSK-PROM clinical feasibility and acceptability

The structured feedback workshop involved four patients, six physiotherapists, one manager and two researchers. Patients felt the MSK-PROM together with the EQ-5D-5L successfully captured relevant domains and was acceptable and feasible for use in clinical practice. The discussion of the potential mis-uses of the measure highlighted: the MSK-PROM is not considered appropriate for asymptomatic conditions or in the context of preventative treatment, prior to the development of symptoms. In one case, the patient described how the tool helped to de-construct and explain the impact of her symptoms to her physiotherapist over the course of treatment. Strong endorsement was provided by the other patient representatives. Clinicians also felt the MSK-PROM was feasible and added value to the consultation by monitoring changes over time. Some further minor improvements to the tool’s readability and comprehension were suggested and at the end of the workshop participants were unanimously in favour of recommending the use of the MSK-PROM as fit for purpose to clinical colleagues or fellow patients.

## Discussion

We have developed a brief patient reported outcome measure (PROM) to monitor health status across a range of musculoskeletal pain disorders and validated key psychometric properties with patients accessing physiotherapy. The new MSK-PROM has six items (including independence, severity of the one thing that is most difficult, work interference, understanding how to deal with the symptoms, ability to do activities/roles that matter, and overall impact) and is designed so that it can be used alongside the EQ-5D-5L in routine clinical practice to assess priority outcome domains.

Having appropriate clinical tools to capture the impact from fluctuating musculoskeletal symptoms is vital to help patients to better manage and monitor their own health[5]. Until now a brief and feasible multi-dimensional clinical tool capturing the key outcomes which matter to individuals with musculoskeletal pain and musculoskeletal clinicians and services has not been available. This is the first instrument specifically developed to address the need for musculoskeletal services to report their healthcare outcomes using a combination of generic and condition specific PROMs.

Having identified that the widely used EQ-5D-5L already captures a number of the priority health outcomes for musculoskeletal patients, a particular strength of our approach has been to reduce patient and clinician burden by designing the MSK-PROM to complement this existing tool. The study results demonstrated that the MSK-PROM responsiveness (i.e. within group effect size and standardised response mean) is twice as large as the EQ-5D-5L, despite being similarly brief and including the same Likert response scale. In addition, patients and clinician feedback confirmed the MSK-PROM’s brevity and partnership with the EQ-5D-5L was particularly valued. In this study there was a deliberate decision to determine the optimal single item to capture each construct identified in the consensus process, using the views of patients and clinicians (face validity) rather than statistical testing. This approach was considered to be more consistent with the study objective of developing a brief patient/user determined scale for use in clinical practice rather than a new research measure. Further research is now needed to compare the MSK-PROM responsiveness in other settings and with other condition specific outcome measures. In addition, it is possible that whilst this instrument seeks to be a bespoke measure for patients with musculoskeletal conditions, many of the domains identified seem highly relevant for patients with other long-term conditions and future research could explore the use of this tool with other patient groups.

The strengths of this study include strong patient and clinician involvement, the large validation cohort embedded within routine physiotherapy practice, and positive endorsement both locally and nationally through an online survey with clinicians and patients. Limitations include a substantial loss to follow-up in the cohort study and the differences in age identified between responders and non-responders. The relatively low three-month follow-up rate of 54% was anticipated as our method of follow-up was fully embedded within routine practice in order to provide a realistic estimate for future physiotherapy clinical audits using the MSK-PROM.

Further research is now required to explore the ability of the MSK-PROM to describe variability in outcomes of healthcare across different services and explore its potential for benchmarking musculoskeletal physiotherapy service performance using appropriate statistical adjustment for clinical case-mix. Generic methods to case-mix adjust PROMs data have been published[30] but specific methodology for case-mix adjustment in musculoskeletal populations is still in relative infancy with further research required[21]. In the Netherlands and the UK, PROMs data are being mandated by purchasers of musculoskeletal healthcare in combination with patient experience measures for accountability purposes[31] and therefore in the future more data are likely to become available. For example, the Royal Dutch Society for Physical Therapy has commissioned a national four-year programme with a series of pilot projects to stimulate the use of PROMs in physiotherapy practice and begin collecting a central source of musculoskeletal service performance information. This trend may soon be followed in the UK, although the integrated use of PROMs on a wide scale will need to overcome identified barriers including establishing a culture of routine data collection, and overcoming fears about how PROMs data may be used by different stakeholders with conflicting interests[32,33].

Having confirmed the MSK-PROM is reliable, responsive and is acceptable and feasible for use in routine practice, we believe it is ready for use in routine musculoskeletal physiotherapy practice. The Keele MSK-PROM tool is freely available for use from the following website http://www.keele.ac.uk/pchs/disseminatingourresearch/researchtools/keelemsk-promtool/ and is recommended for use alongside the EQ-5D-5L in order to capture the key outcome domains for patients with musculoskeletal pain conditions.

## Supporting Information

S1 Text Musculoskeletal Case-mix Descriptors. (PDF)

S2 Text Survey Feedback Comments. (PDF)

S3 Text The Keele MSK-PROM Tool. (PDF)
