# Supplementary material for: Development and Validation of the Keele Musculoskeletal Patient Reported Outcome Measure (MSK-PROM)
Source: PLoS One. 2015 Apr 30;10(4):e0124557. doi: 10.1371/journal.pone.0124557 (PMC4415910; doi:10.1371/journal.pone.0124557)
Supplement: S1 Text — (PDF) [file pone.0124557.s001.pdf]

Musculoskeletal Case-mix Descriptors

This questionnaire is about the health problem for which you are seeking treatment from this service. We would like to ask you now and in the future about your symptoms and about how you are doing. Please answer every question.

1. How old are you (years)? ☐ 2. Gender: M ☐ F ☐ 3. Postcode ☐

4. Have you come for treatment from: Your GP or nurse ☐ A&E (casualty) ☐ Other ☐
Self-referral ☐ Consultant ☐

5. Which **PART of your BODY** is the **main** problem for which you are seeking treatment? (tick 1)

Shoulder	☐	Head	☐	Hip	☐
Elbow	☐	Neck	☐	Knee	☐
Wrist	☐	Back	☐	Ankle/foot	☐
Hand	☐	Widespread pain	☐	Other	☐

6. In the last 3 months, how many times have you seen your **GP** for this main problem? ☐

7. For **HOW LONG** have you had your **CURRENT** episode of this main problem?

Number of Weeks ☐ Months ☐ Years ☐

8. How much do you expect your condition will **RESPOND** to treatment in this service?

It will get worse — It will stay the same — It will be cured

0 ☐ 1 ☐ 2 ☐ 3 ☐ 4 ☐ 5 ☐ 6 ☐ 7 ☐ 8 ☐ 9 ☐ 10 ☐ ☐

9. Do you feel that your **problem is terrible** and that **it is never going to get any better?**

Completely disagree — Completely agree

0 ☐ 1 ☐ 2 ☐ 3 ☐ 4 ☐ 5 ☐ 6 ☐ 7 ☐ 8 ☐ 9 ☐ 10 ☐ ☐

10. What is your current **work** situation?

I am **retired** ☐
I am **not in** paid work, because of my health ☐
I am **not in** paid work, but **not** because of my health (e.g. student) ☐
I am **in work** and have **not** had time off work because of my health ☐
I am **in work** having had time off in the last 3 months ☐ → ☐
I am in work but am currently **on sick leave** because of my health ☐ → ☐

How many days off, in the last 3 months?

11. How would you rate your **GENERAL HEALTH** and well-being for your age?
Please do this by giving a number from 0 to 100 ☐
(where 0= poor health, and 100 = perfect health).
