# Supplementary material for: Development and Validation of the Keele Musculoskeletal Patient Reported Outcome Measure (MSK-PROM)
Source: PLoS One. 2015 Apr 30;10(4):e0124557. doi: 10.1371/journal.pone.0124557 (PMC4415910; doi:10.1371/journal.pone.0124557)
Supplement: S2 Text — (PDF) [file pone.0124557.s002.pdf]

S2: Survey Feedback Comments

Patients' comments and the research team response:

1. I have given the term "Patient generated problem" considerable thought and have put it at the bottom of my list, because I would "personally" remove it and replace it with "Knowledge of condition" which does not make you're top ten. The reason for this is that, I believe, that the more that a patient knows about their condition the more able they are to come to terms with it.
Team response: 'knowledge of condition' comes up a number of times and should be considered. The patient generated item shouldn't be removed as it is consistently ranked highly.

2. All the answers listed are relevant. The above answers are what I think would be helpful to be asked over time to see if there was any improvement in any or all of them. Good starting points if people will open up to how they really feel. However, I would like to see sleep, fatigue, anxiety and mood added.
Team response: These domains appear a number of times and need to be considered.

3. For me I think knowing about the condition and how to manage it is more important than confidence to manage the condition, because until I have knowledge and understand the condition I think it is harder to have confidence that it can be managed.
Team response: 'knowledge of condition' appears to be important for consideration

4. The items which are missing are 'full and comprehensive understanding of the condition' & 'options for treatment'.
Team response: Again understanding of the condition is raised and needs to be considered

5. I don't understand what bothersomeness means! I think 'knowledge' is missing, because when I got physio it was important that she clearly explained what was happening to me so I actually understood it.
Team response: Bothersomeness is perhaps best viewed as overall impact.... 'Knowledge' appears many times and should be considered.

6. All the time in moderate pain may be just as unbearable as periods of high-intensity pain. 'Normal' has to be defined in terms of an individual's personal lifestyle: I was told by a doctor that I didn't have a problem as I could walk for a mile, whereas my previous norm was to hike up mountains without pause.
Team response: Interesting point – we should consider a frequency response scale rather than a severity response scale.

7. I don't exactly understand no 2 (pt generated item). It seems no 3 (ability to self-manage) and 8 (self-efficacy) are about the same. Knowledge of condition added should be added to the top 10.
Team response: Knowing how to deal with the condition is consistently raised.

8. Knowledge of condition and sleep should be added
Team response: These 2 domains have been raised consistently and should be considered

9. Professionals rarely listen to what patients are saying or what they want
Team response: This person wants a patient experience measure about being listened to. Our PROM tool will measure change – and being listened to is hard to measure before consultation so will not be included in the PROM.

Clinician and managers' comments and the team's response:

1. Domains 5 (quality of life) and 10 (overall impact) are too vague.
Team response: They are clearly also important domains to patients – so should remain.

2. Sleep is needed
Team response: this appears a number of times, and is likely to be identified in item 2.

3. Work question will not be applicable to all BUT you should include an option for those who are 'Not working'.
Response: This is an important point for consideration. 'Work/daily routine' was added

4. Anxiety and mood is needed
Team response: this domain should be added

5. Sleep is needed
Team response: this domain appears a number of times and should be considered

6. Sleep is needed
Team response: this domain appears a number of times and should be considered

7. An item to capture psychological interference is needed
Team response: a similar domain of anxiety and mood appears a number of times and should be considered

8. The most important thing in my view is to establish the patients' perception of whether they have improved. As Peter Croft says, if you want to know if the patient is better, you ask them. Patient experience is also important. If they had improvements, but the personal cost was high e.g. due to clinicians' attitude etc, would they use the service again?
Team response: Patient experience such as satisfaction is important but is not a PROM measure.

9. I would prefer to have a criteria associated with mood/ depression as one target. The other one is sleep - poor sleeping patterns disturbed by level of the pain massively worsen the whole presentation and exacerbate many factors above.
Team response: This domain comes up a number of times and should be considered

10. I didn't feel that the top 10 listed were in the right order. They are all important but perhaps it is a reflection of the patient groups that I see on a regular basis that I have chosen some of the treatment targets which just missed out on the 10 ten. I would like fear and coping included.
Team response: This person admits that they treat a select group of patients and these domains have not been raised by others.

11. I think mood is important
Team response: A number of people have added mood to their list and so it should be considered.

12. I feel anxiety and fatigue should be added
Team response: These domains come up a number of times. Fatigue is likely to be identified in item 2, and anxiety clearly needs to be considered for inclusion.

13. I would only change domain 10 to include sleep not bothersomeness
Team response: Sleep comes up a number of times, but is likely to be identified by item 2.

14. There is quite a lot of overlap in the domains and achievement in one will result in meeting others e.g. decreasing fear and anxiety may reduce pain or vice versa or an understanding of bio mechanics will help with prevention etc
Team response: This person also wants knowledge/understanding included. We should consider if we can avoid having similar items.
