# Supplementary material for: Development and Validation of the Keele Musculoskeletal Patient Reported Outcome Measure (MSK-PROM)
Source: PLoS One. 2015 Apr 30;10(4):e0124557. doi: 10.1371/journal.pone.0124557 (PMC4415910; doi:10.1371/journal.pone.0124557)
Supplement: S3 Text — (PDF) [file pone.0124557.s003.pdf]

The Keele MSK-PROM for Monitoring Musculoskeletal Health

This questionnaire is about the health problem for which you are seeking treatment from this service.
Place a **tick** in **one** box for each question below to indicate which statement best describes **your view today**.
Each **column** records a different treatment visit.

**Q1. Needing help**		Visit 1	Visit 2	Visit 3	Visit 4	Visit 5	Visit 6
How often do you need help from others because of your symptoms?							
Never	1						
Rarely	2						
Sometimes	3						
Frequently	4						
All the time	5						

**Q2. Work/daily routine**		Visit 1	Visit 2	Visit 3	Visit 4	Visit 5	Visit 6
How often have your symptoms interfered with your normal work/daily routine (including jobs around the house)?							
Never	1						
Rarely	2						
Sometimes	3						
Frequently	4						
All the time	5						

**Q3. Activities and roles**		Visit 1	Visit 2	Visit 3	Visit 4	Visit 5	Visit 6
How often are you prevented from doing activities and roles that matter to you?							
Never	1						
Rarely	2						
Sometimes	3						
Frequently	4						
All the time	5						

**Q4. Severity of worst problem** (e.g. sleep, fatigue, driving)		Visit 1	Visit 2	Visit 3	Visit 4	Visit 5	Visit 6
Think about the one thing you have the most difficulty with. How often are you finding this difficult?							
Never	1						
Rarely	2						
Sometimes	3						
Frequently	4						
All the time	5						

**Q5. Understanding how to deal with symptoms**		Visit 1	Visit 2	Visit 3	Visit 4	Visit 5	Visit 6
How often do you feel unsure about how to deal with your symptoms?							
Never	1						
Rarely	2						
Sometimes	3						
Frequently	4						
All the time	5						

**Q6. Overall impact**		Visit 1	Visit 2	Visit 3	Visit 4	Visit 5	Visit 6
Overall, how often do your symptoms bother you?							
Never	1						
Rarely	2						
Sometimes	3						
Frequently	4						
All the time	5						

Any and all copyrights © in Questions 1-6, their order and layout vest in Keele University (May 2013).
The tool is scored by summing all 6 items together.
